# Role of Alternative Splicing and Polyadenylation in Regulation of Spleen Development

**DOI:** 10.3390/cells15060496

**Published:** 2026-03-10

**Authors:** Jinghao Cui, Rongru Zhu, Mengke Song, Yuanlu Sun, Yu Pang, Ming Tian, Xinmiao He, Di Liu, Xiuqin Yang

**Affiliations:** 1 College of Animal Science and Technology, Northeast Agricultural University, Harbin 150030, China; 15546686313@163.com (J.C.); zhurongruzi@163.com (R.Z.); songmengke1113@163.com (M.S.); sunyuanlu2023@126.com (Y.S.); pangyu@neau.edu.cn (Y.P.); 2 Institute of Animal Husbandry, Heilongjiang Academy of Agricultural Sciences, Harbin 150086, China; tianming@haas.cn (M.T.); haashxm@163.com (X.H.)

**Keywords:** immunological development, polyadenylation site, spleen, transcript isoform, transcription factor

## Abstract

Alternative splicing (AS) and alternative polyadenylation (APA), as post-transcriptional regulatory mechanisms, are involved in various biological processes through the generation of transcript variants. However, genome-wide studies of AS and APA during spleen development are scarce. This study aimed to characterize transcript diversity and changes in transcript isoforms in the spleen at two developmental stages using full-length isoform sequencing integrated with short-read RNA sequencing. We revealed widespread transcript diversity and identified 17,294 unannotated transcripts, most of which originated from known genes in the current pig genome annotation. The top 500 genes with the highest isoform diversity were mainly associated with disease occurrence and immune function, as revealed by Kyoto Encyclopedia of Genes and Genomes enrichment analysis. We also observed changes in major transcript usage and polyadenylation site selection during spleen development. Our results indicated that genes regulated immunological development mainly by switching dominant transcript isoforms rather than altering overall expression levels. In addition, genes exhibited a tendency of age-dependent preference for distal polyadenylation sites. Furthermore, transcription factors important for spleen development were identified, and the regulatory axes MYBL2/WEE1 and E2F1/WEE1 were constructed for the first time using molecular biology techniques. These findings not only refined the current pig genome annotation, but also provided a foundation for exploring the molecular mechanisms responsible for spleen development.

## 1. Introduction

The immune system is responsible for maintaining a healthy body. It monitors and defends against the invasion of various exogenous pathogens or substances and eliminates otherwise normal cells damaged by infection or malignantly transformed cells. Additionally, the immune system is also responsible for repairing and healing tissue damage caused by injury. It is composed of numerous cells, enzymes, and proteins that work together to maintain body health and integrity. Dysfunction of essential components of the immune system results in increased susceptibility to infections, malignancies, and various autoimmune disorders. The spleen is an important immune organ that orchestrates innate and adaptive immune responses. As the largest secondary lymphoid organ, the spleen participates in multiple immunological functions, including cytokine production, pathogen clearance, and cell differentiation, thereby playing a regulatory role in balancing pro- and anti-inflammatory responses [1].

Alternative splicing (AS) and alternative polyadenylation (APA), predominantly present in eukaryotes, are important post-transcriptional regulatory mechanisms of gene expression that diversify the transcriptome and proteome. AS and APA can generate multiple mRNAs from the same pre-mRNA. More than 95% of human multi-exon genes undergo AS, and approximately half of human protein-coding genes undergo APA [2,3,4,5]. These transcript isoforms may differ in coding sequences (CDSs), untranslated regions (UTRs), or polyadenylation sites (PASs). More than 37% of human protein-coding genes can generate multiple protein isoforms through AS and/or APA [6]. Besides producing distinct polypeptides, AS and/or APA can impact RNA stability and mRNA localization, or modulate other RNAs by competing for shared regulators, thereby playing important roles in regulating gene expression [5,7,8].

Recent advances in high-throughput sequencing technologies have provided powerful tools for genome-wide investigation of AS and/or APA in the immune system. Studies have identified AS and/or APA events in various immune cell types, including T cells, monocytes, and macrophages [9,10,11,12]. Together, these findings highlight the broad impact of AS and APA on gene expression in innate and adaptive immune responses. However, the full spectrum of genes regulated by AS and APA in immune cells has not yet been comprehensively characterized. Most existing studies rely on short-read RNA sequencing (RNA-seq) to analyze AS and/or APA at the genome-wide level. Due to relatively short read lengths, RNA-seq cannot reliably reconstruct full-length transcript structures, making accurate and comprehensive characterization of AS and APA difficult. PacBio full-length isoform sequencing (Iso-seq), a direct and robust approach for detecting full-length transcripts, provides a highly accurate and comprehensive view of transcriptomes [13]. To date, relatively few studies have used Iso-seq to investigate AS and APA in the immune system. In this study, we used Iso-seq to identify transcript isoforms in developing spleen tissues of pigs. Our results expanded the diversity of the porcine spleen transcriptome and highlighted the regulatory roles of AS and APA in spleen development, thereby providing a better understanding of post-transcriptional regulation in immune responses.

## 2. Materials and Methods

### 2.1. Samples and Nucleic Acid Isolation

Min pigs were reared at the Animal Husbandry Research Institute of the Heilongjiang Academy of Agricultural Sciences (Harbin, China). All pigs were fed ad libitum under identical environmental conditions. Male pigs at 30 and 90 days of age (*n* = 3 per age group) were selected and slaughtered according to the Chinese national standard for pig slaughtering (GB/T 17236–2019) [14]. After slaughter, spleen tissues were collected immediately and snap-frozen in liquid nitrogen. All animal procedures were approved by the Laboratory Animal Welfare and Ethics Committee of Northeast Agricultural University. Total RNA was extracted using TRIzol reagent (Invitrogen, Carlsbad, CA, USA), whereas genomic DNA was isolated using DNAiso reagent (TaKaRa, Dalian, China).

### 2.2. Iso-Seq Library Construction and Sequencing

Library construction and Iso-seq were performed by Biomarker Technologies (Wuhan, China). Briefly, RNA samples from the three pigs of the same age were pooled in equal amounts to construct Iso-seq libraries. Full-length cDNA was synthesized using the NEBNext Single Cell/Low Input cDNA Synthesis & Amplification Module (New England Biolabs, Gloucester, MA, USA). PCR amplification was performed using PrimeSTAR GXL DNA Polymerase (TaKaRa), and the products were purified using AMPure PB magnetic beads (PacBio, Menlo Park, CA, USA). Qubit 3.0 (Life Technologies, Foster, CA, USA) and an Agilent 2100 Bioanalyzer (Agilent, Santa Clara, CA, USA) were then used to measure the concentration and size of the products. Primers and polymerase were then bound to the DNA library using the Binding Kit (PacBio). After a second purification with AMPure PB magnetic beads, sequencing was performed on the PacBio Sequel II platform.

### 2.3. PacBio Data Processing

Circular consensus sequence (CCS) reads were generated from raw data with the CCS producer using the parameters --min-rq 0.9 min-passes 3-j 6 min-length 200. Lima (v2.1.0) was used to remove cDNA primers and barcodes. IsoSeq3 (v3.4.0) was used to remove poly(A) tails and identify full-length nonchimeric (FLNC) reads. SMRT Link software (v10.1) was used to cluster FLNC reads into unique consensus isoforms. Minimap2 (v2.20-r1061) was used to align the high-quality consensus isoforms (accuracy > 99%) to the reference genome (*Sus scrofa* 11.1_release109). The cDNA_Cupcake (v28.0.0) was used to remove reads with identity < 0.9 or coverage < 0.85 and to merge reads differing only at the 5′ end. Because of the incomplete nature of the reference genome, some Iso-seq reads extended beyond the reference sequence at the 3′ end, 5′ end, or both. To verify these long sequences, the reference genome was first optimized using RNA-seq data assembled with StringTie (v2.1.6; parameters: -eB -G). Specifically, when multiple overlapping RNA-seq reads were mapped to the terminal regions of the reference sequence, these reads were used to extend the reference. Among RNA-seq reads with terminal nucleotides, those with the shortest 5′ ends were used to define transcription start sites, whereas those with the longest 3′ ends were used to determine transcription termination sites. Iso-seq-derived long reads were then validated against the optimized reference genome. Redundant reads were further filtered using the SQANTI3 (v5.1.1) filtering script to improve alignment accuracy. BUSCO (v3.0.2) was used to analyze the transcriptome completeness. The protein-coding potential of transcripts was assessed using SQANTI3 (v5.1.1).

### 2.4. RNA Sequencing and Data Analysis

A total of six RNA-seq libraries were constructed using the same samples as those used for Iso-seq (30- and 90-day-old pigs, *n* = 3 per age group). Libraries were prepared using the Hieff NGS Ultima Dual-mode mRNA Library Prep Kit for Illumina (Yeasen, Shanghai, China) following the manufacturer’s protocols and purified using Hieff NGS DNA Selection Beads (a superior alternative to AMPure XP). Sequencing was performed by Biomarker Technologies on the Illumina NovaSeq 6000 platform (Illumina, San Diego, CA, USA).

Clean reads were obtained after quality control and filtering of raw data. Reads were mapped to the reference genome (*Sus scrofa* 11.1_release109) using HISAT2 (v2.1.0; parameters: --dta -p 6 --max-intronlen 5000000). StringTie (v2.2.1; parameters: --merge -F 0.1 -T 0.1) was used for assembly under default settings. Genes and transcripts with counts per million (CPM) ≥ 1 in at least three libraries of one group were kept for further analysis. Gene and transcript expression levels were quantified as fragments per kilobase of transcript per million mapped reads (FPKM) using StringTie. Differential expression analysis was conducted using DESeq2 (v1.30.1; default settings: test = “Wald,” fitType = “parametric”), which fit a negative binomial distribution model and performed hypothesis testing using the Wald test. Differentially expressed genes (DEGs) were identified based on |log_2_FoldChange| ≥ 1 and *p* < 0.05. Venn diagrams and heat maps were generated using online tools (https://www.bioinformatics.com.cn/). Gene Ontology and Kyoto Encyclopedia of Genes and Genomes (KEGG) enrichment analyses were performed as described previously [15].

### 2.5. Transcript Diversity Analysis

Astalavista (v3.2) was used to analyze basic AS events in each sample, including exon skipping (ES), intron retention (IR), alternative 3′ splice sites (A3SS), alternative 5′ splice sites (A5SS), and mutually exclusive exons (MEEs). The transcript with the highest expression level and expressing at least > 10 higher than the one with the second highest level was defined as the major transcript (MT) for each gene. Changes in MT expression between 90 and 30 days were calculated as the ratio of the difference in expression levels at 90 days versus 30 days to the expression level at 30 days. Alluvial plots were generated using the OmicShare online tools (https://www.omicshare.com/tools; accessed on 11 June 2025). Protein–protein interaction (PPI) network analysis was conducted using the STRING database (v12.0; https://cn.string-db.org/), and Cytoscape (v3.9.1) was used to visualize networks with interaction scores greater than 0.7.

The number of PASs per gene was calculated by identifying PASs in each transcript. At the same time, TAPIS pipeline (v1.1.3; https://bitbucket.org/comp_bio/tapis/overview; accessed on 15 June 2025) was used to process FLNC reads to identify APA sites. Genes with more than one PAS identified by both methods were defined as APA genes. For each APA gene, the distance between the first and last PAS was defined as the UTRR. Transcripts with PASs located downstream of the midpoint of the UTRR were defined as distal PAS isoforms (M), whereas those upstream were defined as proximal PAS isoforms (m). The expression ratio of M to m isoforms was calculated; genes with ratios > 1 were classified as M genes, whereas those with ratios < 1 were classified as P genes. Polyadenylation signal motifs were predicted using MEME (https://meme-suite.org/meme/; accessed on 10 July 2025).

### 2.6. Transcription Factor Analysis

Transcription factors (TFs) were predicted using AnimalTFDB 4.0 (http://bioinfo.life.hust.edu.cn/AnimalTFDB4/#/; accessed on 12 November 2023), and their target genes were identified through the Harmonizome database (https://maayanlab.cloud/Harmonizome/; accessed on 6 April 2025). TF–target interaction networks were constructed, and key subnetworks were identified using the Edge Percolated Component algorithm implemented in the cytoHubba plugin of Cytoscape (https://cytoscape.org/; accessed on 6 April 2025).

### 2.7. Plasmids and Mutagenesis

Reverse transcription (RT) was performed using the PrimeScript II 1st Strand cDNA Synthesis Kit (TaKaRa). The 3′-UTRs of zinc finger CCCH domain-containing protein 14 (*ZC3H14*), delta-like protein 1 (*DLL1*) and receptor expression-enhancing protein 5 (*REEP5*) with different lengths were amplified from spleen cDNA using CloneAmp HiFi PCR (TaKaRa) and inserted into the pGL3-Promoter vector at the *Xba*I site using In-Fusion Snap Assembly Master Mix (TaKaRa). The full-length CDSs of MYB proto-oncogene like 2 (*MYBL2*) and E2F transcription factor 1 (*E2F1*) were amplified from spleen cDNA using 2× Taq Master Mix (Vazyme, Nanjing, China) and subcloned into the pCMV-HA vector at the *Eco*RI and *Bam*HI restriction sites using T4 DNA ligase (TaKaRa) to generate the overexpression plasmids pCMV-HA-MYBL2 and pCMV-HA-E2F1, respectively. The *WEE1* promoter was amplified from genomic DNA using 2× Taq Master Mix (Vazyme) and cloned into pGL3-Basic at the *Bam*HI and *Hind*III sites using T4 DNA ligase (TaKaRa) to generate the wild-type (WT) reporter construct. Based on the WT plasmid, putative binding sites were deleted using overlap extension PCR to generate mutant type (Mut) reporter constructs, as described previously [16]. Briefly, overlapping fragments containing mutated base pairs at the 5′ ends were amplified using high-fidelity PrimeSTAR Max DNA Polymerase (TaKaRa) and then fused by PCR. The resulting products were subcloned into pGL3-Basic as described earlier. Three mutants targeting *E2F1* binding sites (Mut-1, Mut-2, and Mut-3) and one mutant targeting the *MYBL2* binding site (Mut-1#) were generated. Primers used for plasmid construction and mutagenesis are given in Table S1.

### 2.8. Dual-Luciferase Reporter Assay

PK-15 cells were cultured as described previously [17]. When the cell density reached 70%, the reporter gene was transfected either alone or in combination with overexpression vectors of MYBL2 or E2F1 using Lipofectamine 8000 (Beyotime, Shanghai, China). At 24 h post-transfection, the cells were collected for luciferase activity detection using the Dual-Luciferase Reporter Gene Assay Kit (Yeasen, Shanghai, China). The Renilla luciferase reporter gene *pRL-TK* was used as an internal reference. Relative luciferase activity was calculated as the ratio of firefly luciferase activity to Renilla luciferase activity.

### 2.9. Real-Time Quantitative PCR

Real-time quantitative PCR (qPCR) was used to validate RNA-seq data. The qPCR was performed using cDNA templates from spleen tissue and the Taq Pro Universal SYBR qPCR Master Mix Kit (Vazyme) on a Roche LightCycler 480 II (Roche, Basel, Switzerland) real-time fluorescence quantitative PCR instrument. The amplification condition was set as follows: 95 °C 10 s, 60 °C 15 s, 40 cycles. The β-actin gene was used as an internal reference. The relative expression levels were calculated using the 2^−ΔΔCt^ method. Additionally, qPCR was performed to measure the overexpression efficiency of MYBL2 or E2F1 vectors using cDNA templates isolated from cells collected at 24 h post-transfection; other procedures were the same as described earlier. Primers used for qPCR are listed in Table S1.

### 2.10. Competitive PCR

Competitive PCR was performed to verify AS events resulting in inner insertion/deletion. Briefly, one primer pair was used to amplify transcripts of the same gene with difference in length. Primer pair spanning the insertion/deletion sequences were designed with primer premier (v5.0) and the sequences are given in Table S1. RT and PCR reactions were performed as described above. The amplification condition was set as follows: pre-denaturation at 95 °C 3 min; denaturation at 95 °C 15 s, annealing at 52~60 °C 15 s, extension at 72 °C 90 s; 30 cycles. The products were detected with 2.5% agarose gel electrophoresis. The products of the same gene were purified separately and subjected to sequencing by GeneSoul Technology (Harbin, China).

### 2.11. Western Blot Analysis

The cells were collected at 48 h post-transfection and lysed with a mixture of protease inhibitor cocktail (PIC; Solarbio, Beijing, China) to extract total protein. Protein concentration was measured using a BCA Protein Assay Kit (Beyotime). Proteins were separated by SDS-PAGE (10% separating gel and 5% stacking gel). After electrophoresis, PVDF membrane transfer was performed. The transferred PVDF membranes underwent protein blocking and were incubated with primary antibodies against WEE1 (1:5000 dilution; Catalog Number: 29474-1-AP; Proteintech, Wuhan, China) at 4 °C overnight, followed by incubation with secondary antibodies (1:20,000 dilution; LI-COR) at 37 °C for 1 h. Finally, the PVDF membranes were detected using a LI-COR near-infrared fluorescence imaging system (LI-COR, Lincoln, NE, USA).

### 2.12. Electrophoretic Mobility Shift Assay

Electrophoretic mobility shift assay (EMSA) was performed as described previously [16]. Briefly, nuclear proteins were isolated from HEK-293T cells using a nuclear extraction kit (Solarbio, Beijing, China). Biotin-labeled probes, unlabeled specific competitors, and mutant competitors were synthesized by General Biol (Hefei, China), and their sequences are listed in Table S1. EMSA was performed using a chemiluminescent kit (Beyotime). Probes were first annealed to form double-stranded oligonucleotides and then incubated with 20 μg of nuclear extracts at room temperature for 20 min. In the competitor group, unlabeled specific probes were incubated for 10 min prior to adding labeled probes. The mixtures were electrophoresed on polyacrylamide gels and transferred onto nylon membranes (Beyotime). The results were visualized using an Azure c300 Gel Imaging System (Azure Biosystems, Dublin, CA, USA). Oligo nucleotide sequences used for EMSA are given in Table S1.

### 2.13. Statistical Analysis

Differences between two groups were analyzed using an unpaired *t*-test in GraphPad Prism 9.5.0 and visualized with the same software. The significance threshold was set at *p* < 0.05. * *p* < 0.05, and ** *p* < 0.01.

## 3. Results

### 3.1. A Large Number of Novel Transcripts Existed in Porcine Spleen Tissue

Iso-seq was performed on spleen tissues from 30- and 90-day-old pigs. A total of 91.13 Gb of data were generated from the two libraries, with an average of 45.6 G per sample. In total, 1,025,905 CCS reads (average length = 1.65 kb) were obtained; among these, 774,093 were FLNC reads, producing 396,516 consensus isoforms with an average length of 1.48 kb. After quality control and filtering with SQANTI3, 396,464 (99.99%) high-quality consensus isoforms, comprising the spleen transcriptome, were retained for further analysis (Table S2).

**Figure 1:**
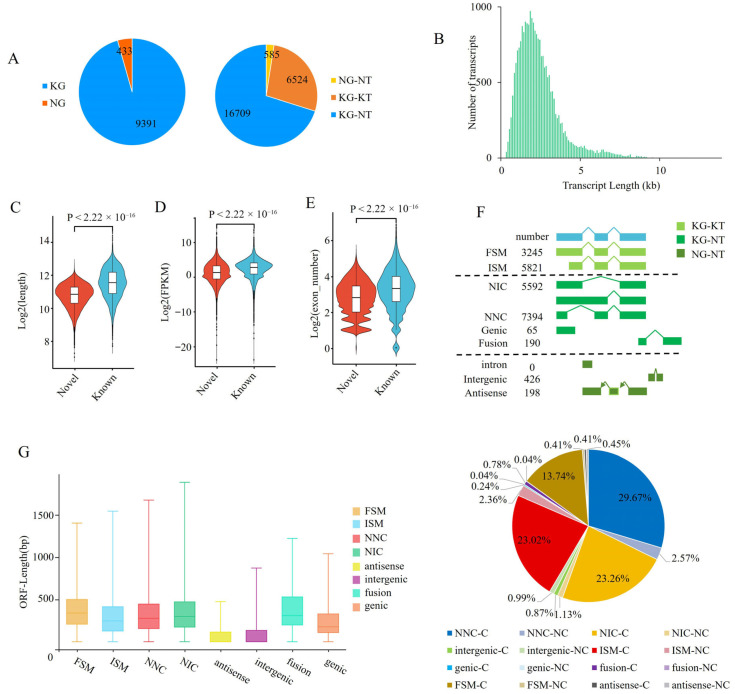
Characterization of novel transcripts. (**A**) Classification of identified genes (**left**) and transcripts (**right**). The number of each category was given in the pie chart. KG, known genes; NG, novel genes; KT, known transcripts; NT, novel transcripts. (**B**) Length distribution of transcripts. (**C**–**E**) Differences between known and novel transcripts in transcript length (**C**), exon number (**D**), and expression level (**E**). (**F**) Subdivision of transcripts based on SQANTI3 classification. FSM, Full splice match; ISM, incomplete splice match; NIC, novel in catalog; NNC, novel not in catalog; C, coding; NC, noncoding. (**G**) Length distribution of open reading frames among different transcript categories.

Alignment to the reference genome (*Sus scrofa* 11.1_release109) showed that the spleen transcriptome consisted of 23,818 transcripts derived from 9824 gene loci, including 9391 annotated genes and 433 novel genes. Among these transcripts, 6524 (3.49 ± 2.15 kb in length, accounting for 27.39%) were known, whereas 17,294 were newly identified (1.92 ± 0.85 kb in length, accounting for 72.61%). Of the novel transcripts, 16,709 originated from known genes and 585 from novel genes (Figure 1A and Table 1 and Table S3). Transcript lengths were predominantly concentrated around 2 kb (Figure 1B). The 17,294 novel transcripts were produced by 7175 genes (accounting for 73.04% of all identified genes), with an average of 2.41 transcripts per gene, indicating extensive transcript diversity within known genes. The results demonstrated that Iso-seq was highly effective for identifying novel transcripts of genes.

Overall, 95.49% of known transcripts (6230 transcripts) were predicted to encode proteins, which was slightly higher than that of novel transcripts derived from known genes (91.47%, *n* = 15,283). In contrast, only 48.38% of the novel transcripts from novel genes (*n* = 283) were predicted to be protein-coding, which was substantially lower than that of novel transcripts from known genes. Additionally, the novel transcripts were shorter than the known transcripts (Mann–Whitney *U* test: *Z* = −57.886, *p* < 0.01; Figure 1C), had fewer exons (Mann–Whitney *U* test: *Z* = −29.088, *p* < 0.01; Figure 1D), and exhibited lower expression levels (Mann–Whitney *U* test: Z = −30.162, *p* < 0.01; Figure 1E). Immune-related genes were abundant in novel transcripts in spleen tissues. The immunoglobulin heavy constant mu (*IGHM*) gene had the largest number of novel transcripts (*n* = 36), whereas only one transcript was annotated in the reference genome. Histone deacetylase 10 (HDAC10), which regulates immunological function through autophagy [18], was associated with 27 novel transcripts. Colony-stimulating factor 1 receptor (CSF1R), a key regulator of monocyte and macrophage proliferation, differentiation, and survival [19], contained 19 novel transcripts. In addition, 14 transcripts of activating transcription factor 6 beta (ATF6) were identified by Iso-seq.

According to SQANTI3 classification, annotated transcripts were subdivided into full splice match (FSM) and incomplete splice match (ISM); novel transcripts from known genes were categorized as novel in catalog (NIC), novel not in catalog (NNC), fusion, and genic; and novel transcripts from novel genes were classified as antisense and intergenic. Overall, the transcriptome consisted of 14.15% FSM, 25.39% ISM, 24.39% NIC, 32.24% NNC, 0.83% fusion, and 3.00% genic, antisense, and intergenic transcripts, with no intron transcripts detected (Figure 1F and Table S4). No significant difference in predicted open reading frame length was observed among FSM, ISM, NIC, and NNC transcripts; however, these categories differed significantly from novel transcripts of novel genes (i.e., antisense and intergenic transcripts) (Figure 1G).

### 3.2. Immune-Related Genes Exhibited Abundant Alternative Splicing in Porcine Spleen Tissue

**Figure 2:**
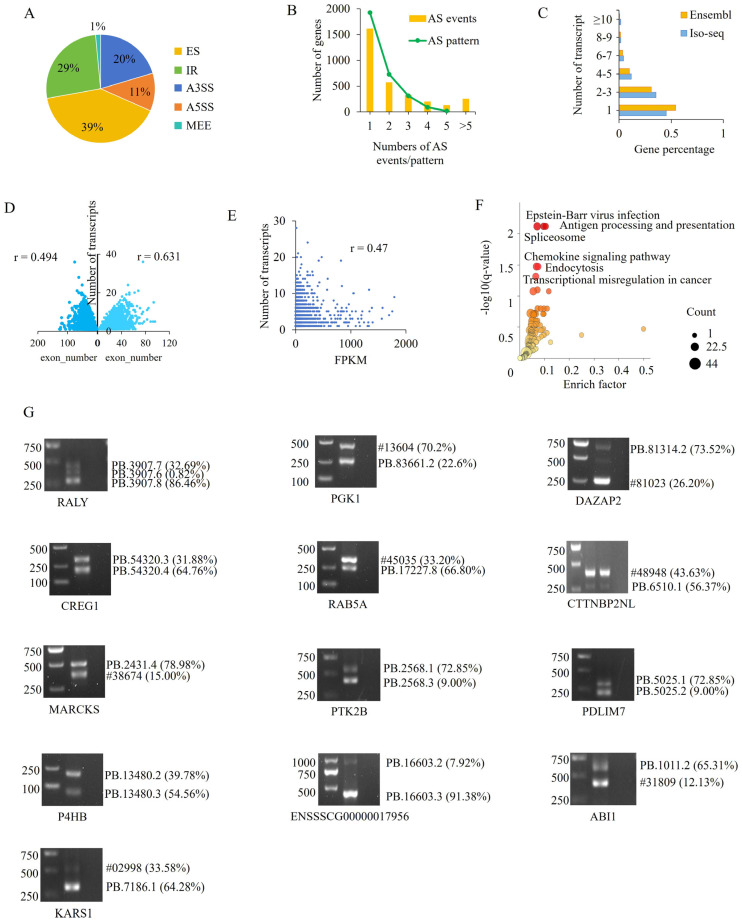
Characterization of alternative splicing (AS) transcripts. (**A**) Classification of AS patterns. ES, exon skipping; IR, intron retention; A3SS, alternative 3′ splice sites; A5SS, alternative 5′ splice sites. (**B**) Distribution of AS events and AS patterns among genes. (**C**) Distribution of transcript numbers among multi-exon genes. (**D**) Correlation between transcript number and exon number. (**E**) Correlation between transcript number and expression level. (**F**) KEGG pathways significantly enriched by the top 500 genes with the highest numbers of transcripts. (**G**) Validation of AS transcripts by competitive PCR. Characters in the right of each gel image indicate transcript name deposited in Ensembl database or assigned by Iso-seq, while those in the bracket indicate the expression proportion of each transcript in the total transcripts of the gene measured with Iso-seq; # represents ENSSSCT000000.

AS is a major contributor to transcriptional diversity. A total of 7467 alternative splicing events, corresponding to 3084 genes (hereafter referred to as AS genes), were identified across the two developmental stages. ES and IR, accounting for 38.26% and 29.82% of total AS events, respectively, were the two most prevalent AS types, followed by A3SS, A5SS, and MEE (Figure 2A). Among these AS genes, 1474 (48.0%) harbored more than two AS events, 1154 genes (37.42%) displayed at least two AS patterns, and 19 genes exhibited all five AS patterns (Figure 2B). A large number of genes (*n* = 5385) were found to produce multiple transcripts, ranging from two to 36, in spleen tissues.

Overall, 9668 and 19,627 multi-exon genes existed in the porcine spleen transcriptome and the reference genome, respectively, of which 55.46% and 51.53% generated more than one transcript, with an average of 3.6 and 3.36 transcripts per gene, respectively. Compared with the reference genome annotation, Iso-seq identified a larger number of genes with multiple transcripts. Notably, the proportion of genes producing ≥10 transcripts was obviously higher than that reported in the reference genome (Figure 2C). Correlation analysis showed that the number of transcripts increased with exon number (*r* = 0.49), and this relationship was more pronounced in highly expressed genes (FPKM > 30; *r* = 0.63) (Figure 2D). In addition, a positive correlation was observed between transcript number and gene expression level (*r* = 0.47) (Figure 2E).

Immune-related genes exhibited particularly abundant transcript diversity. *HDAC10*, heterogeneous nuclear ribonucleoprotein A2/B1 (*hnRNPA2B1*), and nicotinate phosphoribosyl transferase domain containing 1 (*NAPRT*) displayed all five AS patterns, whereas lymphocyte antigen 9 (*LY9*) and TNF receptor-associated factor 7 (*TRAF7*) exhibited four AS patterns. The *IGHM* gene contained the highest number of transcripts. The top 10 genes with the most transcripts were *IGHM*, *HDAC10*, *hnRNPK*, protein phosphatase 1 regulatory inhibitor subunit 2 (*PPP6R2*), methyl-CpG binding domain protein 1 (*MBD1*), *CSF1R*, TRAF3-interacting protein 3 (*TRAF3IP3)*, eukaryotic translation elongation factor 2 (*EEF2*), *hnRNPA2B1*, and regulator of G protein signaling 19 (*RGS19*). Most of these genes are directly or indirectly involved in immune responses. For example, *IGHM* encodes the heavy chain constant region of IgM and plays a crucial role in antigen recognition and immune response [20]. NAPRT has been identified as an endogenous ligand of Toll-like receptor 4 (TLR4) [21], and HDAC10 promotes interferon regulatory factor 3 (IRF3)-mediated antiviral innate immune responses [18].

The top 500 genes with the highest transcript numbers were significantly enriched in six KEGG pathways including Spliceosome, Antigen processing and presentation, Epstein–Barr virus infection, Endocytosis, Chemokine signaling pathway and Transcriptional misregulation in cancer (Figure 2F). Among them, Spliceosome, associated with AS, was the most significantly enriched, and all the remaining ones were associated with disease occurrence and immunological function. To validate the AS events identified by Iso-seq, 13 AS genes were randomly selected for competitive RT-PCR analysis. The expected products were obtained for all selected genes, supporting the reliability of Iso-seq in identifying transcript variants. Additionally, the band intensity of competitive PCR in agarose gel could reflect the quantity of original templates because different transcript templates competitively bind the same primers. We compared the transcript level exhibited by competitive PCR and that by Iso-seq counts (Figure 2G).

### 3.3. Changing the Dominant Isoform Was Important for Regulating Immunological Function

**Figure 3:**
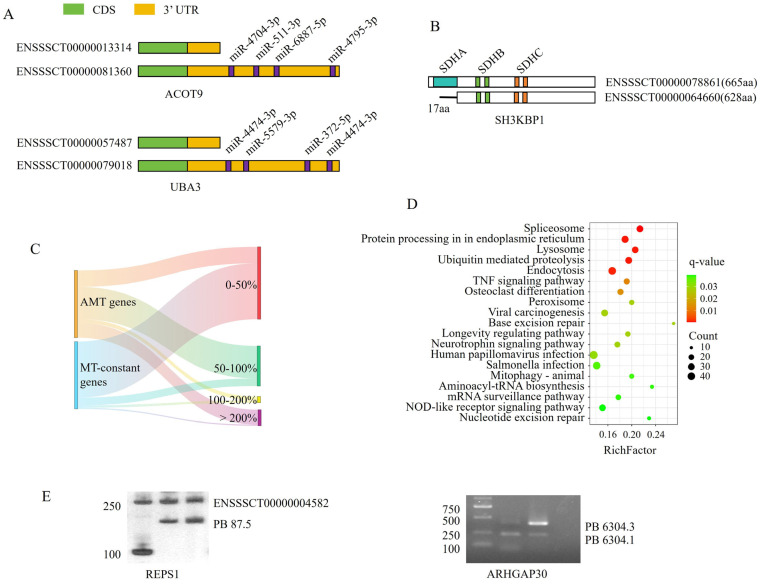
Identification of alternative major transcript (AMT) genes. (**A**,**B**) Changing dominant transcript isoforms resulted in alterations in the regulatory motifs in the 3′ UTR (**A**) or functional domain in polypeptide (**B**). (**C**) Sankey diagram of AMT genes. (**D**) KEGG pathways significantly enriched by AMT genes. (**E**) Validation of AMT genes by competitive PCR.

Transcript abundance was quantified using RNA-seq. A total of 1824 known genes exhibited different MTs between the two developmental stages and were defined as alternative MT (AMT) genes (Table S5). Analysis showed that changing the dominant transcript might have resulted in changes in the regulatory motif in 3′ UTR or in the functional domain in polypeptides (Figure 3A,B). The percentage change in expression at 90 days compared with 30 days was calculated, and genes were grouped into four categories: 0–50%, 50–100%, 100–200%, and >200%. The results demonstrated no obvious difference in expression level, as the proportions of genes in each of the four categories were similar between MT-constant genes and AMT genes (Figure 3C). This indicated that AMT genes regulated spleen development mainly by changing the dominant isoform rather than altering overall expression levels.

**Figure 4:**
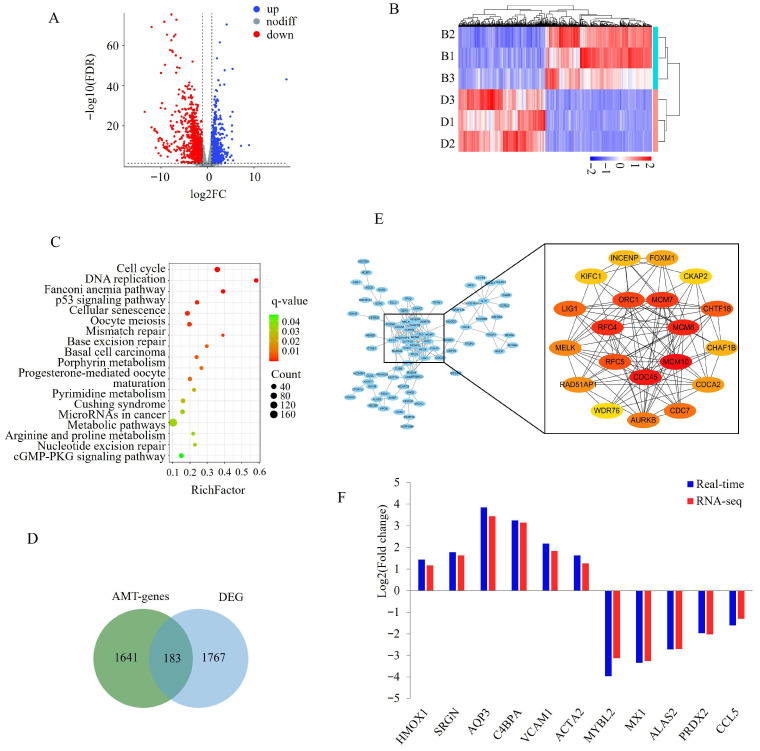
Integrated analysis of Iso-seq and RNA-seq data. (**A**) Volcano plot of RNA-seq data. nodiff = not different. (**B**) Heat map of differentially expressed genes (DEGs) at 90 days compared with 30 days. B and D in the panel indicate 30- and 90-day-old pigs, respectively. (**C**) KEGG pathways significantly enriched by DEGs. (**D**) Venn diagram showing the overlap between AMT genes and DEGs. (**E**) Protein–protein interaction analysis of DE-AMT genes, with node color gradients in the right panel correlated with centrality scores. (**F**) Quantitative PCR validation of RNA-seq data.

AMT genes were significantly enriched in 19 KEGG pathways, and nine out of the 19 pathways were related to health including lysosome, endocytosis, TNF signaling pathway, viral carcinogenesis, longevity regulating pathway, human papillomavirus infection, salmonella infection, mitophagy-animal and NOD-like receptor signaling pathway. Additionally, DNA repair-related pathways, such as nucleotide excision repair and base excision repair, were also significantly enriched. Other enriched pathways were associated with gene expression regulation, including the Spliceosome, protein processing in the endoplasmic reticulum, ubiquitin-mediated proteolysis and mRNA surveillance pathway (Figure 3D). RNA-seq analysis identified 1950 DEGs, including 877 upregulated and 1073 downregulated genes at 90 days compared with 30 days (Figure 4A,B). KEGG enrichment analysis showed that DEGs were mainly involved in cell growth and DNA repair. Four of the top five significantly enriched pathways were directly associated with cell growth, including cell cycle, DNA replication, p53 signaling pathway, and cellular senescence. Among the 18 significantly enriched pathways, Fanconi anemia pathway (ranked third), mismatch repair, base excision repair, and nucleotide excision repair were associated with DNA repair (Figure 4C). Taken together, the enrichment patterns of AMT genes and DEGs suggested that pigs preferentially regulated immunological development through AMT genes.

Integrated analysis of Iso-seq and RNA-seq data identified 183 DE-AMT genes, including 79 upregulated and 104 downregulated genes (Figure 4D and Table S5). PPI analysis revealed that polymerase (DNA) delta 1, catalytic subunit (*POLD1*), minichromosome maintenance complex component 6 (MCM6), replication factor C subunit 4 (*RFC4*), *MCM10*, and *CDC45* were hub genes among the DE-genes (Figure 4E). To validate AMT genes, the RalBP1-associated EPS domain containing 1 (*REPS1*) and Rho-GTPase activating protein 30 (*ARHGAP30*) gene was examined by competitive PCR. Two products were detected, and as expected, the major product differed between the two developmental stages (Figure 3E). Additionally, 11 DEGs were randomly selected for validation by qPCR. All showed expression changes consistent with the RNA-seq results at 90 days compared with 30 days, confirming the reliability of the RNA-seq data (Figure 4E).

### 3.4. Genes Preferentially Used Distal Polyadenylation Sites

**Figure 5:**
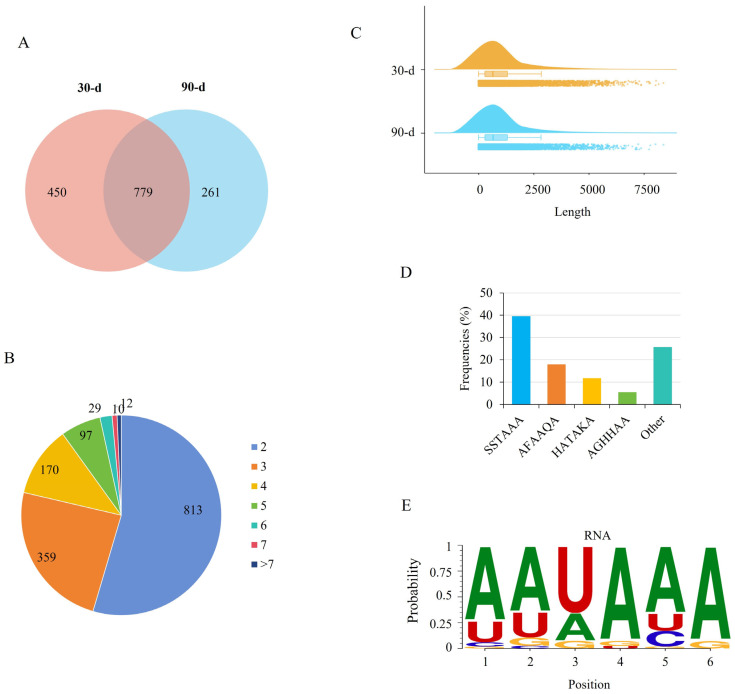
Identification of alternative polyadenylation (APA) sites. (**A**) Statistics of APA genes. (**B**) Distribution of genes by the number of polyadenylation sites. (**C**) Length distribution of 3′ untranslated regions among APA genes. (**D**) Frequency of polyadenylation signal motifs. S = A, C or T; F = A or G; Q = C or T; H = A or T; K =C, G or T. (**E**) Sequence of the most prevalent polyadenylation signal.

APA was analyzed for known genes, which accounted for the vast majority (95.59%) of the genes identified. Multiple PASs were first identified, yielding 17,736 PASs corresponding to 4917 genes based on TAPIS analysis, with an average read support of 8.99 per site. After further positional filtering, 1490 APA genes with 4220 unique PASs were retained, including 3028 PASs (corresponding to 1229 genes) at 30 days and 2554 PASs (corresponding to 1040 genes) at 90 days. Among these, 779 genes exhibited APA in both developmental stages (Figure 5A). The number of PASs per gene ranged from 2 to 12, with most genes harboring two PASs (Figure 5B and Table S6). SERPINE1 mRNA binding protein 1 (SERBP1) and ENSSSCG00000013788 contained the highest number of PASs (12 each). The 3′-UTR lengths of APA transcripts were mainly distributed between 200 and 300 bp, with no significant difference in length distribution between the two stages (Figure 5C). Analysis of polyadenylation signal motifs identified five hexameric sequence motifs, including SSTAAA (S = A, C, or T), AFAAQA (F = A or G; Q = C or U), HATAKA (H = A or U; K = C, G, or T), AGHHAA, and others (Figure 5D). Among these, AATAAA was the most prevalent motif, consistent with its canonical role in polyadenylation (Figure 5E).

**Figure 6:**
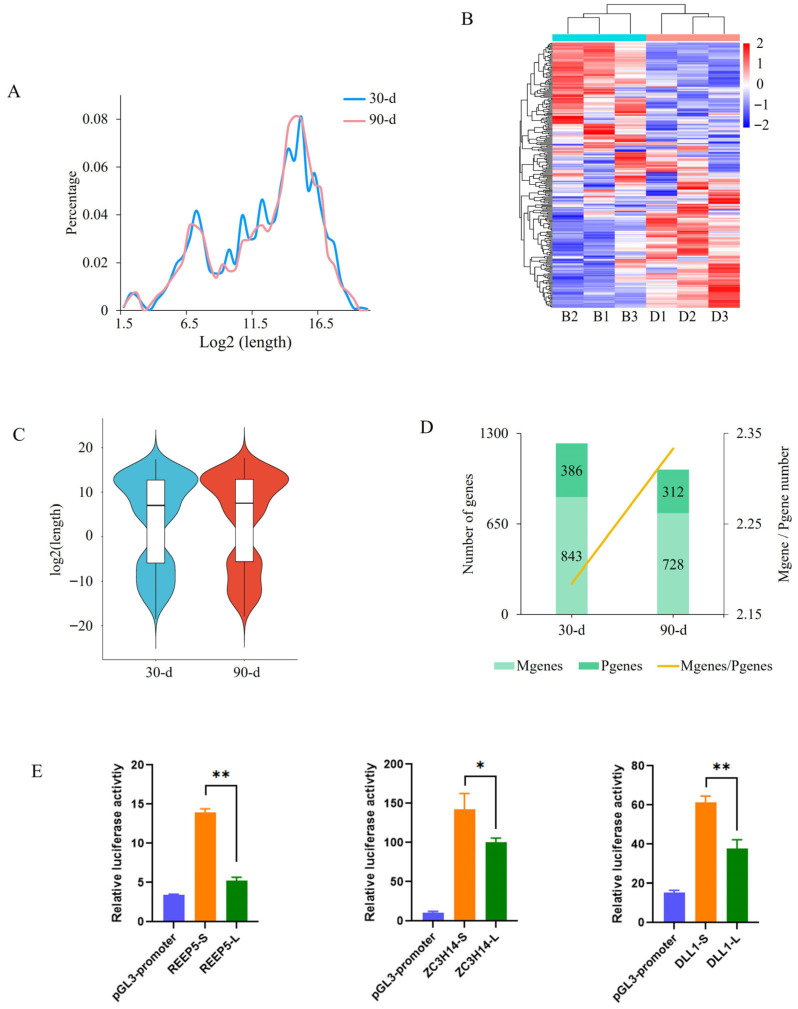
Identification of alternative polyadenylation (APA) sites. (**A**) Length distribution of the region between the first and last polyadenylation sites (PASs). (**B**) Heat map of genes showing different major PAS usage between the two stages. B and D in the panel represent 30- and 90-day-old pigs, respectively. (**C**) Length distribution of major PASs along the 3′-UTR. (**D**) Distribution of PAS usage in APA transcripts. (**E**) Dual-luciferase reporter analysis of the effect of 3′-UTR length on gene expression. L, Long 3′-UTR; S, short 3′-UTR. *, *p* < 0.05; **, *p* < 0.01.

The region between the first and last PAS was designated as UTRR. The overall length of UTRR showed no difference between the two stages; however, the most prevalent UTRR length at 30 days was 41.26% longer than that at 90 days (Figure 6A). The PAS with the highest usage frequency was defined as the major PAS (mPAS) for each gene. Among the 779 genes exhibiting APA in both stages, most (530) had a single mPAS, whereas 249 genes showed a shift in mPAS between the two stages (Figure 6B and Table S6). Relative to the midpoint of the UTRR (1/2 UTRR), most mPASs were located in distal regions in both stages (Figure 6C). To further analyze PAS utilization in the spleen, the genes were classified according to the expression levels of transcripts using different PASs. Transcripts with PASs located downstream of 1/2 UTRR were defined as distal PAS isoforms (M), whereas those upstream were defined as proximal PAS isoforms (m). Genes with an M/m expression ratio > 1 were designated as M genes, and those with a ratio < 1 were designated as P genes. More than 70% of genes in each stage were M genes. The ratio of the number of M genes to P genes increased from 2.18 at 30 days to 2.33 at 90 days (Figure 6D), indicating a tendency toward increased distal PAS usage during spleen development.

To validate the regulatory effects of APA, three APA genes, *ZC3H14*, *REEP5*, and *DLL1*, each harboring two 3′-UTRs of different lengths, were selected for dual-luciferase reporter assays. Two of the genes, *ZC3H14* and *REEP5*, are AMT genes. All tested 3′ UTRs significantly regulated luciferase expression. Also, 3′-UTR length was associated with regulatory activity: in all three genes, the shorter 3′-UTR exhibited higher activity than the longer one (Figure 6E). These results confirmed an important role of 3′-UTR length in regulating gene expression.

### 3.5. Identification of TFs Important for Spleen Development

**Figure 7:**
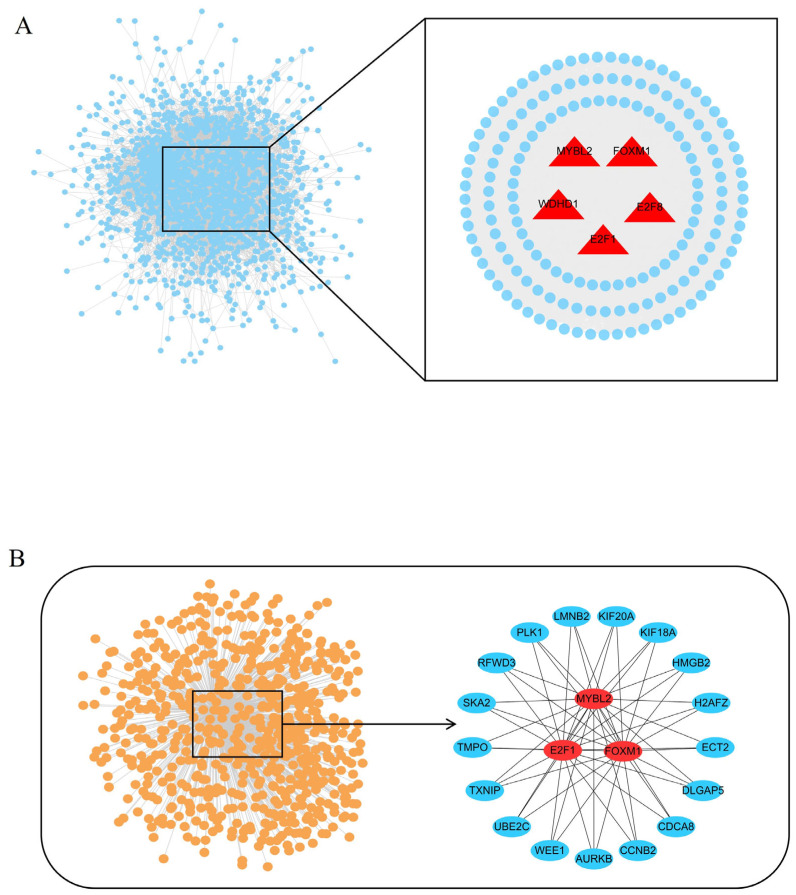
Characterization of transcription factors (TFs) important for spleen development. (**A**) Protein–protein interaction analysis of DE-TFs and DEGs (**left**) and a subnetwork of five key DE-TFs with five or more nodes (**right**). (**B**) Interaction network of the five DE-TFs and their target DEGs predicted by Harmonizome (**left**), and the subnetwork of three hub DE-TFs (**right**).

A total of 93 DE-TFs were identified by RNA-seq. A PPI network was constructed using DE-TFs and DEGs to evaluate the relationships between them. E2F8, Forkhead box protein M1 (FOXM1), WD repeat and HMG-box DNA binding protein 1 (WDHD1), E2F1, and MYBL2 were found to have the highest connectivity, with each node interacting with five or more partners (Figure 7A and Table S7). The target genes of these five TFs were predicted among the DEGs using the online Harmonizome (https://maayanlab.cloud/Harmonizome/; accessed on 12 November 2023) database, and the interaction network was visualized using Cytoscape (v3.8.2). FOXM1, E2F1, and MYBL2 emerged as hub TFs within this network (Figure 7B), suggesting their important roles in regulating spleen development.

**Figure 8:**
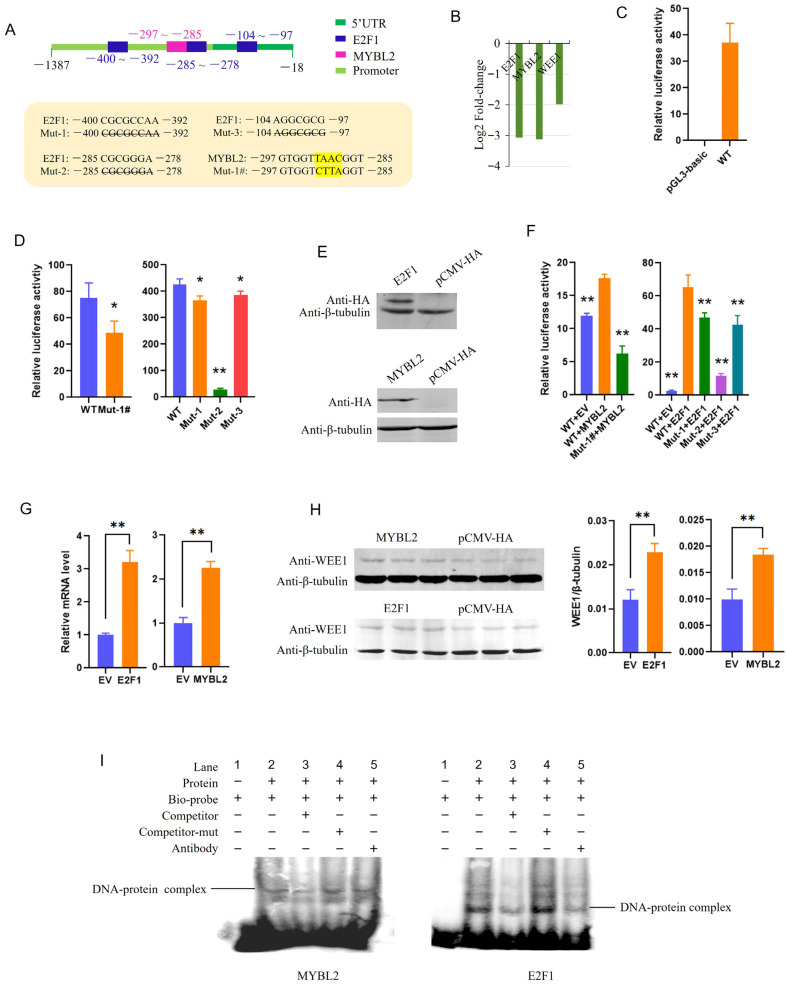
Confirmation of the regulatory roles of transcription factors E2F1 and MYBL2 in WEE1 gene expression. (**A**) Schematic structure of the WEE1 promoter and the putative binding sites of E2F1 and MYBL2. Binding sites and the position are given in the schematic structure, while sequences for each motif in the wild-type and mutant-type (Mut) promoters are given below. Sequences shown with strikethrough were deleted, and those highlighted in yellow were mutated in the Mut reporter constructs. (**B**) Log2Fold-change in E2F1, MYBL2 and WEE1 in 90- compared to 30-day-old pigs as revealed with RNA-seq. (**C**) Characterization of the promoter activity of WEE1 gene with dual-luciferase reporter analysis. WT, wild-type reporter gene. (**D**) Deletion of each putative binding site significantly decreased promoter activity. (**E**) Expression efficiency of plasmids overexpressing MYBL2 or E2F1 as revealed with Western blotting. (**F**) MYBL2 or E2F1 regulated reporter gene expression via the putative binding sites. (**G**) Overexpression of MYBL2 or E2F1 upregulated the mRNA level of WEE1 in PK-15 cells. (**H**) Overexpression of MYBL2 or E2F1 upregulated the protein level of WEE1 in PK-15 cells. (**I**) Direct binding of MYBL2 or E2F1 to the motifs as revealed by EMSA. EV, empty vector. * *p* < 0.05, ** *p* < 0.01, compared with WT reporters in Panels (**D**,**F**).

WEE1 was randomly selected to validate the regulatory relationship presented in Figure 7B. Three putative E2F1 binding sites and one MYBL2 binding site were present in the promoter of the WEE1 gene as revealed with online software Jaspar (https://jaspar.elixir.no/) and TFDB4 (https://guolab.wchscu.cn/AnimalTFDB4/#/; accessed on 12 November 2023) (Figure 8A). RNA-seq revealed that MYBL2, E2F1 and WEE1 were all downregulated significantly at 90 days compared with 30 days (Figure 8B), preliminarily indicating the existence of a regulatory axis between MYBL2/E2F1 and WEE1. It was first shown that the WT plasmids could robustly drive luciferase gene expression (Figure 8C), indicating that the inserted fragment was the promoter of the WEE1 gene. The luciferase activity of the WEE1 promoter was significantly reduced (*p* < 0.01) after mutation of each of the four putative binding sites (Figure 8D), demonstrating the involvement of these sites in WEE1 gene expression. Plasmids overexpressing E2F1 or MYBL2 were then successfully constructed (Figure 8E). Co-transfection of the WEE1 reporter gene with E2F1 or MYBL2 significantly increased luciferase activity (*p* < 0.01), whereas deletion or mutation of the binding sites weakened the promoting effects of MYBL2 or E2F1 (Figure 8F). Additionally, the effects of MYBL2 or E2F1 on endogenous WEE1 gene expression were examined. The results showed that both mRNA and protein levels of WEE1 significantly increased after overexpression of each TF (*p* < 0.01) (Figure 8G,H). Furthermore, EMSA was used to analyze the binding of MYBL2 and E2F1 to the predicted sites. Clear nuclear protein-probe complexes were observed; the complexes were weaker in the competitor group, and the probes were not competitive after mutation. Additionally, antibody addition reduced the intensity of the complexes (Figure 8I). These results indicated that both TFs regulated WEE1 expression by directly binding to its promoter.

## 4. Discussion

In this study, we used Iso-seq together with RNA-seq to characterize full-length transcripts and depict AS and APA in porcine spleen tissues in two developmental stages. Considerable transcript isoform diversity was observed in spleen tissues, largely due to the identification of numerous novel transcripts from known genes in the reference genome. Full-length transcripts from previously unannotated genes were also detected. Notably, AMT and PAS usage differed between the two developmental stages, indicating changes in transcript diversity. Additionally, we found that with increasing age, genes have a tendency to use distal PASs, suggesting the involvement of 3′-UTR lengthening in spleen growth and development. Together, these results enriched the porcine reference genome and highlight the roles of AS and APA in the growth and development of spleen tissues.

Our data represented the most comprehensive full-length transcriptome analysis of porcine spleen reported to date. We demonstrated that the current porcine genome annotation remained incomplete, and a substantial proportion of genes might produce novel transcripts. Our data revealed novel exons, polyadenylation sites, and even entire genes not currently annotated in existing porcine databases. Importantly, some genes exhibited period-specific transcripts, further increasing transcriptome diversity. By using Iso-seq, numerous novel isoforms were identified in sheep, killifish and series of plants, and AS and APA events were shown to be surprisingly more prevalent than previously estimated in primates [13,22,23]. Even in humans, Iso-seq revealed that the complexity of the transcriptome is underestimated significantly [13]. These results showed that Iso-seq is a useful tool for identifying AS transcripts and that the transcriptome is complex and dynamic.

As only two developmental stages of spleen tissues were used for identifying transcript diversity in this study, it is reasonable to expect that additional novel genes and transcripts may have existed in a breed-, tissue-, and stage-specific manner. Thus, transcript diversity in pigs likely far exceeded our current understanding, and many transcripts remained to be identified. We also observed relatively low expression levels of novel transcripts and a moderate correlation between transcript numbers and gene expression levels. Previous studies have shown that highly expressed genes are prone to incorrect splicing, potentially leading to misassembled transcripts [24]. Thus, some transcripts may contain by-products of highly expressed genes, although RT-PCR amplification has validated the reliability of the identified AS transcripts/events. Nevertheless, this resource substantially improves our understanding of the expressed transcript repertoire in porcine spleens.

We further demonstrated the significant contribution of AS and APA to transcript isoform diversity in the spleen. ES was the most common form of AS in the spleen, followed by IR, in both developmental stages, consistent with previous analyses on longissimus thoracis in pigs and donkeys [15,25]. In contrast, some studies using Iso-seq reported IR as the predominant AS type in porcine mixed skeletal muscles tissues, and human and mouse cerebral cortexes [26,27]. Evidence in C. elegans, mice and humans has shown that IR increases with age and is implicated in Alzheimer’s disease [28,29,30]. Studies also showed an increase in exon inclusion/intron retention in 210-day-old pigs compared to 7-, 30-, 60- and 90-day-old pigs [15]. The results obtained here might be caused by developmental stages as piglets were used. Also, the results may reflect differences in species, or tissues. Although genome-wide studies have revealed the importance of AS in transcript diversity in pigs, APA has received comparatively limited attention. This study showed that APA occurred in a substantial proportion of genes, with an average of ~3.55 PASs per APA gene, which was higher than expected. A previous study reported that 32.86% of PAS-associated genes in porcine skeletal muscles contained more than one PAS [31]. Additionally, single-cell polyadenylation analyses identified 20,222 PASs corresponding to 9454 genes in human and mouse cells, among which 43.4% harbored two or more PASs [32]. Collectively, these findings emphasized the importance of APA in transcript diversity and transcriptome complexity.

APA can affect either the coding sequence or the length of the 3′-UTR, thereby influencing the regulation of biological processes. Changes in global 3′-UTR landscapes have been reported during oocyte meiotic maturation and oocyte-to-zygote transition of embryos in pigs [33,34]. Global APA patterns also differ between Large White and Tongcheng pigs in response to PRRSV infection [35]. Moreover, tissue-specific PAS usage has been identified between fast and slow muscle types, and is associated with muscle development in pigs [31]. Furthermore, numerous studies have associated APA with development, differentiation and diseases in humans [36,37,38]. These studies demonstrate the functional relevance of APA from a genome-wide perspective. The present study further showed that the differential length of 3′-UTR caused by APA contributed to gene regulation, as validated using molecular biological techniques, thereby providing experimental evidence for the regulatory importance of APA. Variations in 3′-UTR length can increase or decrease binding motifs for miRNAs or RNA-binding proteins, and further analysis of these interactions can elucidate the underlying mechanisms by which APA regulates gene expression.

Additionally, we demonstrated developmental changes in transcript isoform abundance and PAS usage preferences in spleen tissues, highlighting an important role for transcript diversity in immune system regulation and development. Immune-related genes not only exhibited extensive transcript diversity but also included many AMT genes involved in health-associated pathways. By integrating Iso-seq and RNA-seq data, we showed that the changes in transcript abundance represented an important mechanism underlying immunological development. Transcript variants are frequently involved in regulating biological activities. For example, an alternative transcript of peroxisome proliferator-activated receptor γ (PPARγΔ5) regulates adipogenesis by impairing the function of WT PPARγ [39]. Similarly, a transcript isoform of forkhead box p1 (FOXP1)-ES promotes the expression of pluripotency-associated genes, such as nanog homeobox and octamer-binding transcription factor 4, and plays a vital role in reprogramming of somatic cells to induced pluripotent stem cells [40,41]. Consistent with these findings, our results demonstrated the functional importance of AS and APA through the generation of transcript isoforms during spleen development. It should be noted that PAS usage preference between ages was just analyzed on Iso-seq data. Iso-seq was performed on pooled RNA and no replicates were set at each developmental stage. This part of the results is descriptive rather than statistically powered. Nevertheless, we have identified numerous splicing variants involved in spleen development. Our future studies will focus on elucidating the specific roles of alternative splicing variants in selected genes, which will further clarify the regulatory mechanisms of AS and APA in spleen development.

Finally, we characterized essential TFs regulating spleen development and constructed the regulatory network using their target DEGs. It is interesting that in the core subnetwork composed of TFs FOXM1, E2F1, and MYBL2, all 17 targets are related to cell proliferation. In particular, WEE1, CCNB2, CDCA8, ECT2, SKA2, RFWD3, and PLK1, etc., are key regulators of cell proliferation and/or cell cycle [42,43,44]. Here, 30- and 90-day-old piglets, that is, piglets in the nursery and growth phases, were used. During this period, not only does the pig’s immune system gradually mature, but its spleen also undergoes rapid growth. Cell proliferation is the basis for growth and development. Numerous DEGs related to cell proliferation/cell cycle were identified as targets of hub TFs, consistent with the growth and development characteristics of pigs during this period. Among the DEGs targeted by hub TFs, WEE1 was randomly selected for validating the core subnetwork constructed. WEE1 is a serine/threonine protein kinase and can inactivate cyclin-dependent kinase 1 via phosphorylating it at tyrosine14 and tyrosine15 residues, thereby playing a crucial role in cell cycle regulation and cell proliferation. Specifically, it is a G2/M phase checkpoint kinase and controls entry into mitosis [43]. Here, WEE1 is downregulated in spleens of 90- compared to 30-day-old pigs, consistent with its negative role in regulating cell cycle progression and the rapid growth of these pigs. By confirming the E2F1/WEE1 and MYBL2/WEE1 axis with molecular biological techniques, it is shown that the regulatory network, especially the core subnetwork, constructed with TFs and their targets is reliable. Therefore, we provided a series of TF-target pairs important for regulating spleen development.

## 5. Conclusions

We constructed a full-length transcriptome of porcine spleen in two developmental stages using PacBio Iso-seq. Our data revealed that AS and APA were widespread and many transcripts remained unannotated in the current porcine genome, indicating tissue- and stage-specific characteristics of AS and/or APA. We also demonstrated changes in MT abundance and PAS usage during spleen development. Importantly, we showed that the changes in MTs was an important mechanism regulating immunological development, and genes showed a tendency to use distal PAS with age. Additionally, key TFs involved in spleen development were identified. Collectively, these findings not only enriched the current pig genome annotation, but also provided a valuable basis for exploring the molecular mechanisms underlying spleen development.

## Figures and Tables

**Table 1:** Summary of the spleen transcriptome obtained by Iso-seq.

	Number (%)
Unique genes	9824
Annotated genes	9391 (95.59%)
Novel genes	433 (4.41%)
Transcripts	23,818
Known transcripts (%)	6524 (27.39%)
Novel transcripts	17,294 (72.61%)
Genes with >1 isoform	5385 (54.81%)
Genes with ≥10 isoforms	169 (1.72%)

## Data Availability

The transcriptome datasets presented in this study have been deposited to the National Genomics Data Center (NGDC, https://ngdc.cncb.ac.cn/) with the dataset accession number CRA030038.

## Supplementary Materials

The following supporting information can be downloaded at: https://www.mdpi.com/article/10.3390/cells15060496/s1, Supplementary Materials Table S1: Primers and oligonucleotides; Supplementary Materials Table S2: Statistics of Iso-seq data; Supplementary Materials Table S3: Transcripts identified by Iso-seq; Supplementary Materials Table S4: Classification of transcripts with SQANTI; Supplementary Materials Table S5: Characterization of alternative major transcripts; Supplementary Materials Table S6: Characterization of polyadenylation sites; Supplementary Materials Table S7: Characterization of transcription factors.

## Abbreviations

The following abbreviations are used in this manuscript:

AS	Alternative splicing
APA	Alternative polyadenylation
CDSs	Coding sequences
UTRs	Untranslated regions
PAS	Polyadenylation site
RNA-seq	RNA sequencing
Iso-seq	Isoform sequencing
CCS	Circular consensus sequence
FLNC	Full-length non-chimeric
FPKMs	Fragments per million transcripts
DEGs	Differentially expressed genes
KEGG	Kyoto Encyclopedia of Genes and Genomes
ES	Exon skipping
IR	Intron retention
A3SSs	Alternative 3′ splicing sites
A5SSs	Alternative 5′ splicing sites
MEEs	Mutually exclusive exons
MT	Major transcript
PPI	Protein–Protein Interaction
TFs	Transcription factors
EPC	Edge Percolated Component
qPCR	Real-time quantitative PCR
EMSA	Electrophoretic mobility shift assay
IGHM	Immunoglobulin heavy constant mu
HDAC10	Histone deacetylase 10
CSF1R	Colony-stimulating factor 1 receptor
ATF6	Activating transcription factor 6 beta
FSM	Full splice match
ISM	Incomplete splice match
NIC	Novel-in-catalog
NNC	Novel-not-in-catalog
hnRNP	Heterogeneous nuclear ribonucleoprotein
LY9	lymphocyte antigen 9
TRAF	TNF receptor-associated factor
MBD1	Methyl-CpG binding domain protein 1
TRAF3IP3	TRAF3-interacting protein 3
EEF2	Eukaryotic translation elongation factor 2
RGS19	Regulator of G protein signaling 19
TLR4	Toll-like receptor 4
IRF3	Interferon regulatory factor 3
AMT	Alternative MT
REPS1	RalBP1 associated EPS domain containing 1
SERBP1	SERPINE1 mRNA binding protein 1
mPAS	Major PAS
