# Supplementary material for: Role of Alternative Splicing and Polyadenylation in Regulation of Spleen Development
Source: Cells. 2026 Mar 10;15(6):496. doi: 10.3390/cells15060496 (PMC13025901; doi:10.3390/cells15060496)
Supplement: Supplementary file 1 [file cells-15-00496-s001.zip › Supplementary Table S1 Primers and oligonucleotides.pdf]

Supplementary Table S1-1 Primers for qPCR verification

| Primer name | Gene ID | Sequence (5'-3') | Length |
|---|---|---|---|
| *VCAM1* | ENSSSCG00000006862 | F: GGCTAGCGGATGCAGGTGTAT<br>R: ACCCCGATGGCAGGTATTATTA | 169 bp |
| *HMOX1* | ENSSSCG00000039745 | F: TACCGCTCCCGAATGAACAC<br>R: CGAGGGTCTCTGGTCCTTAGT | 150 bp |
| *SRGN* | ENSSSCG00000023374 | F: TGAATCCAACAGAATCCTACCT<br>R: CAATTTCATTTAGGGAGCCAC | 146 bp |
| *C4BPA* | ENSSSCG00000015662 | F: AGTGTTCTGGCTATGGTTTGGTT<br>R: TTGCTCACAGTCTTCGGGGT | 113 bp |
| *AQP3* | ENSSSCG00000040513 | F: CCTTATCCTCGTGATGTTTGG<br>R: AGCACATAGCGAAGGTCACAG | 179 bp |
| *MX1* | ENSSSCG00000012077 | F: CACATCTGTAAAACTCTGCCCCT<br>R: CCTCTCCTTGTATCAAAGCAGTG | 181 bp |
| *PRDX2* | ENSSSCG00000013736 | F: TTGACTCTCAGTTCACCCACCT<br>R: TCTTCCTTCAGCACACCATAATC | 133 bp |
| *CCL5* | ENSSSCG00000017705 | F: CCACACCCTGCTGTTTTTCC<br>R: CACACCTGGCGGTTCTTTCT | 130 bp |
| *ACTA2* | ENSSSCG00000010447 | F: CCTGGTGTGTGACAATGGTTC<br>R: TTCCTCTTTTGCTTTGGGCTT | 170 bp |
| *ALAS2* | ENSSSCG00000012347 | F: TCAGCCCTACTCTTCTCTTCCT<br>R: CTCCACTATTGCGGATACCTT | 139 bp |
| *WEE1* | ENSSSCG00000013411 | F: CAGTGCTTGGACAACATTCTCAT<br>R: GTAGTTTTCACTTATGGCATCAGC | 125 bp |
| *MYBL2* | ENSSSCG00000007366 | F: GAAACCAGCCTACCCTACAAGT<br>R: TACTCAGGTCACACCAGGCGT | 127 bp |
| *E2F1* | ENSSSCG00000007268 | F: CGCATCTACGACATCACCAAC<br>R: AGCAGATGCAGCTGTGTGGTAC | 206 bp |
| β-actin | ENSSSCG00000007585 | F: CCAGGTCATCACCATCGG<br>R: CCGTGTTGGCGTAGAGGT | 158 bp |

Supplementary Table S1-2 Primers for AS events verification

| Primer name | Gene ID | Sequence (5'-3') | Length |
|---|---|---|---|
| *KARS1* | ENSSSCG00000002704 | F: CAGGCGGCTGAAGTTAAAGTG<br>R: CGATGAAGTGAGTGAGCGAGAT | 496/316 bp |
| *CTTNBP2NL* | ENSSSCG00000006781 | F: TCCAGAGCGCAGGGAAAAG<br>R: TTGAGAGTGGGTTTGTGCAGAC | 396/212 bp |
| *PDLIM7* | ENSSSCG00000040507 | F: AGCACAGAGTAAACCGCAGAAG<br>R: GCTTGATTTTTTCAGGTGCTCC | 317/197 bp |
| *PTK2B* | ENSSSCG00000009664 | F: CGACCTCTGCCCACCTATTCT<br>R: CTGCCTCAGCCACTGGTAATC | 532/406 bp |

| | | | |
|---|---|---|---|
| *ABI1* | ENSSSCG00000024127 | F: CAGAGACCAAGGACGCACAG<br>R: CATACCAGCCATCGTCGTTCT | 583/409 bp |
| *RALY* | ENSSSCG00000007275 | F: GCCTTCTCCCTCCTTCCTTC<br>R: ACGGCTGTGTTGAGGTTTCC | 431/367 bp |
| *PGK1* | ENSSSCG00000012440 | F: CTCAACAACATGGAGATTGGCA<br>R: CCAGTGCTCACATGGCTGAC | 443/265bp |
| *DAZAP2* | ENSSSCG00000022674 | F: CCTTGCATCTTCCTCAGGCT<br>R: ATACCCTCCTTCCACCAGCA | 663/253bp |
| *P4HB* | ENSSSCG00000039148 | F: GCAAGAACGTCTTCGTGGAG<br>R: CACCCAACTGATCCCAGACG | 153/81bp |
| *MARCKS* | ENSSSCG00000037803 | F: AGTGCGGCTACAAATCTGGG<br>R: CGCGTCGCCGTTTACTTTC | 477/378bp |
| *ENSSSCG00000017956* | ENSSSCG00000017956 | F: TACGGACAGCTCACCTTTGG<br>R: TGGGTGTTGGAACAGATGCT | 963/427bp |
| *CREG1* | ENSSSCG00000006308 | F: GTCTTATCTCTCAGCGACGGC<br>R: CTAGGACCCAGATGTTCGTGA | 340/220bp |
| *RAB5A* | ENSSSCG00000037653 | F: GGAGCACAAGCAGCCATAGT<br>R: GCGTGGGTTCAGTAAGGTCTA | 343/249bp |
| *ARHGAP30* | ENSSSCG00000006372 | F: TCACCCGTGGCCTTGAGTAC<br>R: CAGAGACAGGTCATCCAAGGGT | AMT verification |
| *REPS1* | ENSSSCG00000004146 | F: CGCCATCAATGCCATCACTA<br>R: ATGTTTTCACAAGCAATGGGC | AMT verification |

Supplementary Table S1-3 Primers for APA verification

| Primer name | Gene ID | sequence 5'-3' | Length |
|---|---|---|---|
| *ZC3H14*-L | ENSSSCG00000002426 | F:AGATCGCCGTGTAATTCTAGAGTGAATGACATCCAGTCCTACCTG<br>R:GCCGGCCGCCCCGACTCTAGACAAACACGCCTCAAATGTTTTAA | 315bp |
| *ZC3H14*-S | ENSSSCG00000002426 | F:AGATCGCCGTGTAATTCTAGAGTGAATGACATCCAGTCCTACCTG<br>R:GCCGGCCGCCCCGACTCTAGAGCAATAAATTTCATATTATGAAAGCAAT | 179bp |
| *DLL1*-L | ENSSSCG00000027726 | F:AGATCGCCGTGTAATTCTAGACCGAGGTGTAAAGTGGAGGTGA<br>R:GCCGGCCGCCCCGACTCTAGATGTTCATAATATTTATTTTGGAAAAATATTT | 688bp |
| *DLL1*-S | ENSSSCG00000027726 | F:AGATCGCCGTGTAATTCTAGACCGAGGTGTAAAGTGGAGGTGA<br>R:GCCGGCCGCCCCGACTCTAGACCACCTAGAAAAACCACATTTTAGTATC | 425bp |

| | | | |
|---|---|---|---|
| *REEP5*-L | ENSSSCG00000022048 | F:AGATCGCCGTGTAATTCTAGACCTAAACCACTGGCTGGATG<br>R:CCGGCCGCCCCGACTCTAGAAAAATAAAGTTTAATAAGCT | 1588bp |
| *REEP5*-S | ENSSSCG00000022048 | F:AGATCGCCGTGTAATTCTAGACCTAAACCACTGGCTGGATG<br>R:CGGCCGCCCCGACTCTAGAACCTAATTGTTATCTGCAAG | 1432bp |

Note: The underline indicates the enzyme site of XbaI.

Supplementary Table S1-4 Primers used for vector construction and site-directed mutagenesis

| Name | Gene ID | Sequence (5'-3') | Purpose |
|---|---|---|---|
| *MYBL2* | ENSSSCG00000007366 | F:CGGAATTCGGATGTCTCGGCGGACG<br>R: GGGGTACCCGCCAAGTCCCTAGGACAAGAT | CDS region amplification |
| *E2F1* | ENSSSCG00000007268 | F:TATGGCCATGGAGGCCCGAATTCTGAGCGCCATGGCCGTGGC<br>R:ATCCCCGCGGCCGCGGTACCATCAGAAATCCAGGGGGGTGAG | CDS region amplification |
| *WEE1* | ENSSSCG00000013411 | F: GGGGTACCAGAGCGGTGTGGCAGTTTA<br>R: CCAAGCTTGGCGAAGGACCGGAGAAT | Promoter region amplification |
| *WEE1*-MT-1# | ENSSSCG00000003398 | F: ACGTGGTCTTAGGTCGCGGGAAGCCGC<br>R: CGCGACCTAAGACCACGTGAGTGCCCAG | *MYBL2* site mutation |
| *WEE1*-MT-1 | ENSSSCG00000003398 | F: AGCGAGTTAATCGCGTAGCCGATCCCTC<br>R: TACGCGATTAACTCGCTGGCTCCGCCCAC | *E2F1* site mutation |
| *WEE1*-MT-2 | ENSSSCG00000003398 | F: TTAACGGTAGCCGCGGAGCCCGAACCT<br>R: TCCGCGGCTACCGTTAACCACGTGAGT | *E2F1* site mutation |
| *WEE1*-MT-3 | ENSSSCG00000003398 | F: AAGCCGCTCCCAGTCGCACGTGGCG<br>R: CGACTGGGAGCGGCTTCGGCGGCGGGC | *E2F1* site mutation |

Note: The underline indicates the enzymes sites of EcoRI, BamHI or HindIII.

Supplementary Table S1-5 Oligonucleotides used for electrophoretic mobility shift assay

| | Primer name | Sequence（5'-3'） |
|---|---|---|
| Biotin labeling probe | Bio-MYBL2-F | bio-GCACTCACGTGGTTAACGGTCGCGG-bio |
| | Bio-MYBL2-R | bio-CCGCGACCGTTAACCACGTGAGTGC-bio |
| | Bio-E2F1-F | bio-GTTAACGGTCGCGGGAAGCCGCGGAG-bio |
| | Bio-E2F1-R | bio-CTCCGCGGCTTCCCGCGACCGTTAAC-bio |
| competition probe | MYBL2-F | GCACTCACGTGGTTAACGGTCGCGG |
| | MYBL2-R | CCGCGACCGTTAACCACGTGAGTGC |
| | E2F1-F | GTTAACGGTCGCGGGAAGCCGCGGAG |
| | E2F1 -R | CTCCGCGGCTTCCCGCGACCGTTAAC |
| Mutation probe | MYBL2-M-F | GCACTCACGGTTCGTCTGGTCGCGG |
| | MYBL2-M-R | CCGCGACCAGACGAACCGTGAGTGC |

| | | |
|---|---|---|
| | E2F1-M-F | GTTAACGGTTTACTATAGCCGCGGAG |
| | E2F1-M-R | CTCCGCGGCTATAGTAAACCGTTAAC |
